# Influence of Elemental Carbon (EC) Coating Covering nc-(Ti,Mo)C Particles on the Microstructure and Properties of Titanium Matrix Composites Prepared by Reactive Spark Plasma Sintering

**DOI:** 10.3390/ma14010231

**Published:** 2021-01-05

**Authors:** Anna Biedunkiewicz, Paweł Figiel, Dariusz Garbiec, Aleksei Obrosov, Mirosława Pawlyta, Witold Biedunkiewicz, Przemysław Pruss, Krzysztof Rokosz, Rafał Wróbel, Steinar Raaen, Sabine Weiß, Dmitry Bokov

**Affiliations:** 1 Department of Materials Technology, Faculty of Mechanical Engineering and Mechatronics, West Pomeranian University of Technology Szczecin, 19 Piastow Avenue, 70-310 Szczecin, Poland; anna.biedunkiewicz@zut.edu.pl (A.B.); pruss.przemyslaw@zut.edu.pl (P.P.); 2 Łukasiewicz Research Network–Metal Forming Institute, 14 Jana Pawla II Street, 61-139 Poznan, Poland; dariusz.garbiec@inop.lukasiewicz.gov.pl; 3 Department of Metallurgy and Materials Technology, Faculty 3: Mechanical Engineering, Electrical and Energy Systems, Brandenburg University of Technology Cottbus—Senftenberg, 17 Konrad-Wachsmann-Allee Street, 03-046 Cottbus, Germany; aleksei.obrosov@b-tu.de (A.O.); sabine.weiss@b-tu.de (S.W.); 4 Institute of Engineering Materials and Biomaterials, Faculty of Mechanical Engineering, Silesian University of Technology, 18A Konarskiego Street, 44-100 Gliwice, Poland; miroslawa.pawlyta@polsl.pl; 5 Department of Manufacturing Engineering, Faculty of Mechanical Engineering and Mechatronics, West Pomeranian University of Technology Szczecin, 19 Piastow Avenue, 70-310 Szczecin, Poland; witold.biedunkiewicz@zut.edu.pl; 6 Division of Surface Electrochemistry & Technology, Faculty of Mechanical Engineering, Koszalin University of Technology, 15-17 Raclawicka Street, 75-620 Koszalin, Poland; rokosz@tu.koszalin.pl; 7 Department of Catalytic and Sorbent Materials Engineering, Faculty of Chemical Technology and Engineering, West Pomeranian University of Technology Szczecin, 10 Pulaskiego Street, 70-322 Szczecin, Poland; rafal.wrobel@zut.edu.pl; 8 Department of Physics, Norwegian University of Science and Technology, Realfagbygget E3-124 Høgskoleringen 5, NO 7491 Trondheim, Norway; steinar.raaen@ntnu.no; 9 Institute of Pharmacy, Sechenov First Moscow State Medical University, 8 Trubetskaya Street, Bldg. 2, Moscow 119991, Russia; bokov_d_o@staff.sechenov.ru

**Keywords:** nanocomposites, TiMMCs, spark plasma sintering, (Ti,Mo)C/C, EBSD, fracture toughness

## Abstract

This paper describes the microstructure and properties of titanium-based composites obtained as a result of a reactive spark plasma sintering of a mixture of titanium and nanostructured (Ti,Mo)C-type carbide in a carbon shell. Composites with different ceramic addition mass percentage (10 and 20 wt %) were produced. Effect of content of elemental carbon covering nc-(Ti,Mo)C reinforcing phase particles on the microstructure, mechanical, tribological, and corrosion properties of the titanium-based composites was investigated. The microstructural evolution, mechanical properties, and tribological behavior of the Ti + (Ti,Mo)C/C composites were evaluated using X-ray diffraction (XRD), scanning electron microscopy (SEM), energy dispersive X-ray spectroscopy (EDX), electron backscatter diffraction analysis (EBSD), X-ray photoelectron spectroscopy (XPS), 3D confocal laser scanning microscopy, nanoindentation, and ball-on-disk wear test. Moreover, corrosion resistance in a 3.5 wt % NaCl solution at RT were also investigated. It was found that the carbon content affected the tested properties. With the increase of carbon content from ca. 3 to 40 wt % in the (Ti,Mo)C/C reinforcing phase, an increase in the Young’s modulus, hardness, and fracture toughness of spark plasma sintered composites was observed. The results of abrasive and corrosive resistance tests were presented and compared with experimental data obtained for cp-Ti and Ti-6Al-4V alloy without the reinforcing phase. Moreover, it was found that an increase in the percentage of carbon increased the resistance to abrasive wear and to electrochemical corrosion of composites, measured by the relatively lower values of the friction coefficient and volume of wear and higher values of resistance polarization. This resistance results from the fact that a stable of TiO_2_ layer doped with MoO_3_ is formed on the surface of the composites. The results of experimental studies on the composites were compared with those obtained for cp-Ti and Ti-6Al-4V alloy without the reinforcing phase.

## 1. Introduction

In order to obtain a synergy of useful properties that are beyond the reach of titanium and its alloys, titanium-based composites are used, in which the reinforcing phase and the matrix have been properly tailored. The particle reinforcement of titanium and its alloys provides the possibility of significant improvements in mechanical properties [1,2,3,4]. Generally, metal matrix composites are used in wear resistant applications, where high hardness combined with a reasonable level of toughness occurs [5,6,7]. One of the key aspects in which MMCs intrinsic properties may be used is wear resistance and high strength. 

Titanium matrix composites (TiMMCs) have attracted properties such as light-weight, high strength, good corrosion resistance, and excellent biocompatibility. Among the different reinforcements, TiC was widely used, owing to its excellent compatibility with titanium matrix [8,9,10].

The decreasing of the ceramic particle size can result in considerable improvement in the mechanical properties of TMCs, e.g., enhanced strengthening and reduced particle cracking [11,12,13].

The strength of composites is mainly determined by the size of the strengthening phase and the interfacial bonding between the reinforcement and the matrix. The wettability between ceramics and metals is usually poor, and limited bonding between particles and matrix may cause severe problems [14,15,16].

In order to maximize interfacial bonding in TiMMCs, it is necessary to promote wetting and minimize oxide formation. Among the different TiMMCs sintering processes, in situ reactive sintering is a promising method because of its relatively low cost and simplicity. Moreover, in situ reactive processing enables to fabricate high performance TiMMCs because it combines clean interfaces between reinforcement and matrix, and also refinement of the reinforcement. The good interfacial connection (consolidation of the composite) is possible because during the sintering processes, chemical reactions between the matrix and the strengthening phase take place. This approach guarantees a thermodynamically stable composite with clean interphase surfaces, well-consolidated, with maximum strength at low and elevated temperatures.

The powder metallurgy route is commonly adopted to manufacture titanium metal matrix composites. We have introduced an innovative solution for TiMMC interfacial reinforcement, consisting of sintering a mixture of titanium powders and (Ti,Mo)C-type carbide embedded in carbon shell. Carbon shell has an additional function: it protects the carbide from oxidation in storage conditions and various types of process operations. The isomorphous TiC and MoC_1-x_ carbides form a continuous series of solid solutions at high temperatures. Stabilization of cubic MoC can also be achieved by replacing a small amount of Mo atoms by Ti atoms together with the formation of carbon vacancies. Under the sintering process (Ti,Mo)C-type carbide may undergo spinodal decomposition into TiC and MoC forms, and the further consequence may be a reaction of molybdenum carbide with titanium, resulting in the formation of metallic molybdenum, contributing to the stabilization of the β-Ti(Mo) phase. Some of these considerations and studies can be found in the literature [17,18,19,20,21,22].

In this work, the spark plasma sintering (SPS) technique was used for the titanium-based composite manufacturing process. SPS, in detail described in [23], is a constantly developed sintering technique for rapid consolidation of powder materials in minutes as compared to several hours required for conventional sintering. SPS involves simultaneous application of compaction pressure and pulsed DC with a low voltage (up to max. 8–10 V) and high current (from max. 3 kA for small samples with diameter of max. 30 mm to max. 24 kW for large samples with diameter of max. 150 mm), directly to the powder materials. As a result of applied pulsed DC, at the beginning, an electrical discharges is generated in the voids between powder particles and temperature on the surface of the powder particles rises. As a consequence, cleaning effect occurs, and the diffusion starts at lower temperature. Furthermore, with ongoing consolidation, the necks are formed and powder is directly heated by Joule effect [24]. The main difference between SPS and other sintering methods e.g. hot pressing (HP) is that in SPS, a direct heating of the powder takes place.

In previous work [22], we presented the results of research on composites obtained from the same substrates using the incremental technique of selective laser melting (SLM). After the SLM process, the surface composites require further machining treatments due to high roughness and porosity. Such technological problems are less frequently observed in the production of metals and their alloys. Therefore, the authors of this work focused on the SPS technique.

## 2. Materials and Methods

### 2.1. Preparation of Titanium Composites

**Figure 1:**
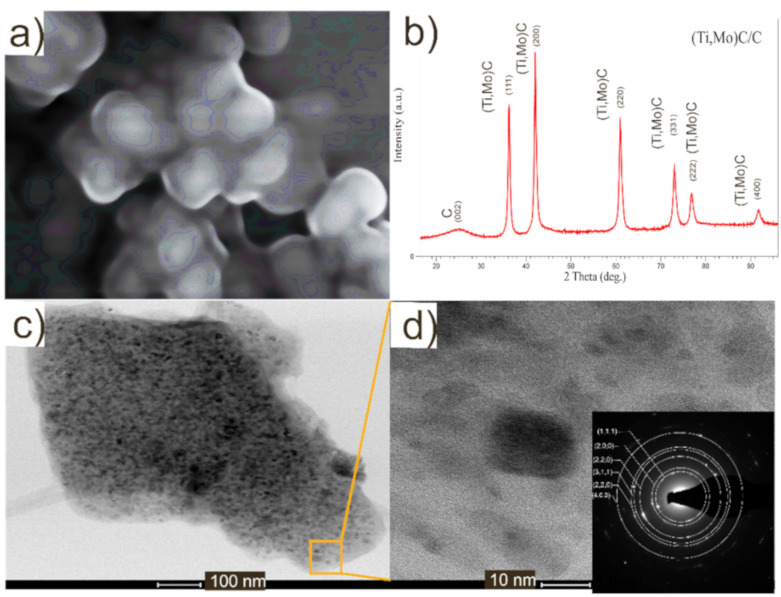
Morphology of the starting material: (**a**) SEM micrograph; (**b**) XRD diffraction pattern; (**c**) and (**d**) TEM micrograph of nc-(Ti,Mo)C/C crystals in carbon shell and corresponding SAED pattern.

One type of cp-Ti (Grade 1) from SLM Solutions (Lübeck, Germany) obtained by gas atomization was employed as starting matrix material. Cp-Ti had a spherical shape and average particle size was around 100 μm. Two types of (Ti,Mo)C nanoparticles (NPs) embedded in the carbon shell were used as reinforcements. The first type marked as nc-(Ti,Mo)C/C consisted of nanoparticles with an average crystallite size of 12 nm and carbon content of about ca. 40 wt %, whereas the second type marked as nc-(Ti,Mo)C were nanoparticles with an average crystallite size of 17 nm and carbon content of ca. 3 wt %. Based on previous research, the molar ratio of Ti/Mo has been determined as 10:1 [22]. The micrographs and XRD pattern of the particles is presented in Figure 1. Nanocrystalline carbides of the (Ti,Mo)C and (Ti,Mo)C/C-type were obtained by a nonhydrolytic sol-gel method [25]. The Ti with the (Ti,Mo)C or (Ti,Mo)C/C powders was milled in argon atmosphere using a planetary mill Pulverisette 4 (Fritsch, Idar-Oberstein, Germany), with use of the WC milling balls in a weight ratio of 10:1 with respect to the powder. For the sintering process, the mixtures of the powders composed of 10 and 20 wt % of the (Ti,Mo)C or (Ti,Mo)C/C powders and the titanium were used. Four types of composites have been manufactured and marked as follows: #1—Ti + 10 wt % (Ti,Mo)C, #2—Ti + 10 wt % (Ti,Mo)C/C, #3—Ti + 20 wt % (Ti,Mo)C, #4—Ti + 20wt % (Ti,Mo)C/C. For comparison, the samples of the alloy of Ti6Al4V and cp-Ti were prepared using the same method and under the same conditions as in the case of the composites. To prepare samples of the cp-Ti and Ti6Al4V alloy, commercial SLM Solutions (Lübeck, Germany) powders were used.

SPS was used to study the effect of the presence of carbon shell on the surface of the (Ti,Mo)C particles distributed in titanium matrix in the consolidation process on the properties of the TiMMCs. For this purpose, an HP D 25/3 (FCT Systeme, Rauenstein, Germany) furnace was used. The following parameters of the sintering process were used: 1300 °C (sintering temperature), 400 °C/min (heating rate), 2.5 min (holding time), 50 MPa (compaction pressure), 5 × 10^−2^ mbar (vacuum). The samples obtained in the process had a cylindrical shape with a diameter of 20 mm and a height of 10 mm.

### 2.2. Structural Characterization

All tests, except for lamellas, were performed on a surface perpendicular to the direction of the applied compaction force of the composite. Specimens for these tests were cut, then ground, and polished using an ATM SAPHIR 550 grinder (Mammelzen, Germany) with a MD-Mezzo 220 disk and MD-Largo discs (Ballerup, Denmark) with the addition of 9 μm diamond suspension. Finishing step was carried out on MD-Chem disks using Al_2_O_3_ suspension with the addition of H_2_O_2_. For morphology investigation, the second group of specimens was sanded with 220 grit paper and electro-polished with Struers LectoPol-5 device (Ballerup, Denmark), with the following parameters: 30–35 V voltage, 16 flow, 20 s time, 1 cm^2^ surface in Struers A3 electrolyte.

The relative density of composites was measured using Archimedes principle. The average size of the (Ti,Mo)C crystallites in the powder was measured by Scherrer and Debye-Hulla methods. The size of crystallites, morphology phase, and chemical composition of the composites were determined with the following techniques: X-Ray diffraction (XRD) using PANalytical PW3040/60 X’Pert Pro apparatus (Malvern, UK) equipped with Cu Kα radiation, the FEI Titan G2 80-300 TEM/STEM microscope equipped with EDS energy dispersive X-ray spectrometer (Hillsboro, OR, USA), used to analyze the structure and morphology of the composites. The lamellas were prepared on SEM/Ga-FIB FEI Helios NanolabTM 600i apparatus (Hillsboro, OR, USA). The specimens were cut in a direction parallel to the direction of applied compaction force, i.e. perpendicular to the surface analyzed by XRD and XPS. The lamellas were cleaned using a plasma cleaner. Composites of (Ti,Mo)C-Ti type containing 10 and 20% carbides, respectively, were subjected to electron backscatter diffraction (EBSD) measurements performed using a MIRA II scanning electron microscope (SEM) from TESCAN (Brno, Czech Republic) equipped with an EDAX EBSD system (TSL OIM Data Collect and TSL OIM Analysis, Mahwah, NJ, USA).

X-ray photoelectron spectroscopy (XPS) studies were performed on SES-2002 (Scienta Omicron AB, Uppsala, Sweden) using Al Kα (hν = 1486.6 eV) X-ray source (18.7 mA, 13.02 kV). Analyses were carried out with a pass energy of 500 eV, the energy step 0.2 eV and time step of 200 ms [26]. For the XPS analyses, XPS tables [27,28] was used and all the binding energy were charge corrected to *C* 1 s at 284.8 eV [27].

### 2.3. Mechanical and Corrosion Tests

The surface roughness of specimens was determined by means of 3D confocal laser scanning microscope of the Keyence VK-X1000 type (Mechelen, Belgium). An average roughness (*R_a_*) was calculated from a randomly selected surface area of 200 × 200 µm^2^ with 5 nm resolution in z-direction. Mechanical properties (hardness and elastic modulus) were measured using nanoindenter UNAT (ASMEC, Radeberg, Germany) at 100 mN. Berkovich diamond indenter was used to perform nanoindentation test by means of the Quasi Continuous Stiffness Method (QCSM). Twenty measurements on each specimen were performed. An X-Y indentation matrix with 5 × 4 measurements separated by the distance of 100 μm from one another was formed. The hardness (*H*) and elastic modulus (*E*) were derived from these load-displacement curves using the Oliver and Pharr method [29]. Fused quartz (SiO_2_) and sapphire (Al_2_O_3_) standard materials were used for calibration before measurements. Fracture toughness was calculated by the radial indentation crack method described in [30] using Vickers hardness tester (HV_1_) with a dwell time of 15 s. Furthermore, elastic recovery was calculated as plastic work/(plastic work + elastic work) to characterize plasticity of the nanocomposites [31].

The corrosion resistance of the samples was examined using open circuit corrosion potential (OCP) and potentiodynamic polarization measurements (PDP) in 3.5 wt % NaCl solution, in natural oxygen containing state at RT. These tests were carried out on the Atlas-Sollich 9833 potentiostat (Rębiechowo, Poland) in a three-electrode system: the tested samples as a working electrode, calomel as a reference and graphite as auxiliary electrodes. The OCP measurements were performed in the corrosion cell during 15 h. The PDP tests were carried out using the following parameters: a scan rate of 0.01 V/s and potential range between −1.5 V to +2.0 V. The corrosion current calculations were performed using Tafel Slope Analysis by means of the AtlasLab software. During the tests, the electrolytes were in natural oxygen containing state in room temperature. 

The tribological (wear) properties of the specimens were studied using the ball-on-disk technique. The tests were performed under ambient dry conditions using TRN tribometer (CSM, Peseux, Switzerland). Alumina ball with a diameter of 6 mm was slid in rotating mode on disc made of tested material. The applied load was 3 N, with linear speed of 60 mm/s and sliding distance of 500 m. Before each test, the specimens and balls were rinsed ultrasonically in acetone. Friction coefficients were determined. DEKTAK 6M type profilometer (Veeco, Billerica, MA, USA) was used to estimate the volume losses (*V*) and wear coefficient (*K*).

## 3. Results and Discussion

Electron backscatter diffraction (EBSD) examination results reveal the microstructure specific features, phase composition, and crystallographic orientation of the grains. Carbides of the TiC type as well as α-Ti, β-Ti and Mo grains were identified. The percentages of the individual phase components are listed in Table 1. The results demonstrate a considerable increase in TiC content in the composites with (Ti,Mo)C/C nanoparticles (specimen #2 and #4).

**Figure 2:**
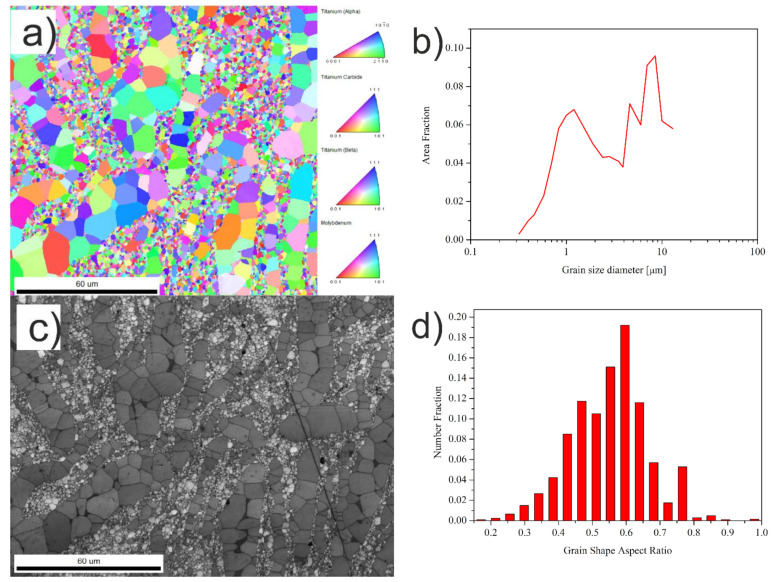
(**a**) EBSD micrograph; (**b**) grain size distribution; (**c**) SEM micrograph; (**d**) grain shape aspect ratio of the Ti + 20 wt %TiMoC composite.

**Figure 3:**
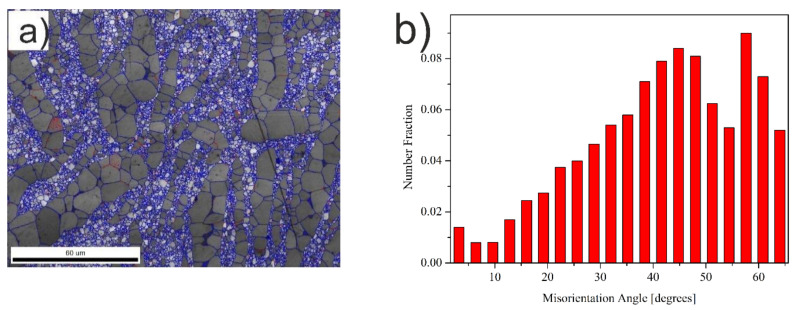
(**a**) Grain boundaries; (**b**) grains misorientation of the Ti + 20 wt %TiMoC composite.

The bimodal microstructure of the Ti + 20wt %TiMoC composite is found (Figure 2a,b). The coarse grains (15–20 μm) of titanium are located between TiC submicron grains and are arranged in the form of bands. The EBSD micrograph presented in Figure 3a revealed the grain boundaries of the Ti + 20 wt %TiMoC composite. The high angle grain boundaries (HAGB, >15°) marked by blue color is the dominant grain boundaries type and make up 94.5% of all grain boundaries. The low angle brain boundaries (LAGB, <15°) marked by red color make up 5.5% of all grain boundaries and are located mainly as a subgrain boundaries in titanium grains. The misorientation of the grains (Figure 3b) increases up to 40–45°. However, the most often misorientation is 55°.

**Figure 4:**
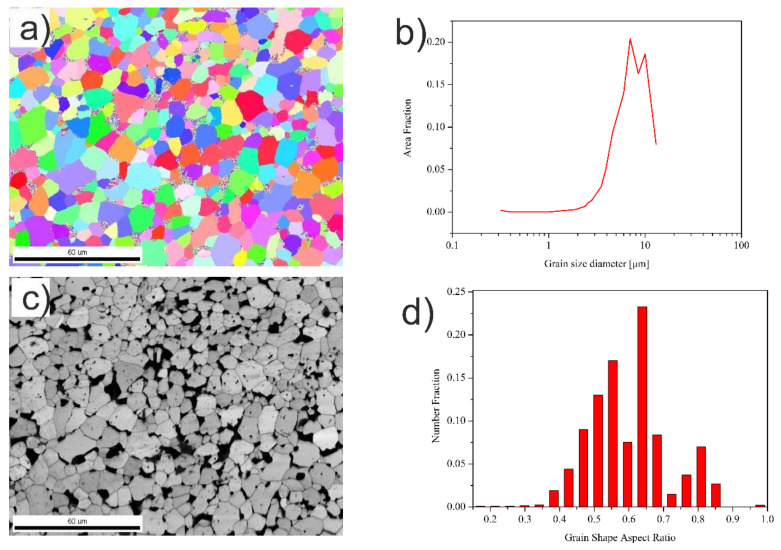
(**a**) EBSD micrograph; (**b**) grain size distribution; (**c**) SEM micrograph; (**d**) grain shape aspect ratio of the Ti + 20 wt %TiMoC/C composite.

In contrast to the Ti + 20 wt %TiMoC (composite #3), the addition of carbon caused a significant evolution of the microstructure. In the Ti + 20 wt %TiMoC/C (composite #4), the uniform microstructure is clearly seen but a lot of pores are visible at the grain boundaries (Figure 4a,c). An average TiC grain size significantly increases in comparison to the composite #3. The most dominant are the coarse grains with average size of 8.5 μm and 12 μm (Figure 4b). In this case, the grains have a slightly different shape aspect ratio within the range 0.40–0.55 and 0.60–0.65. 

**Figure 5:**
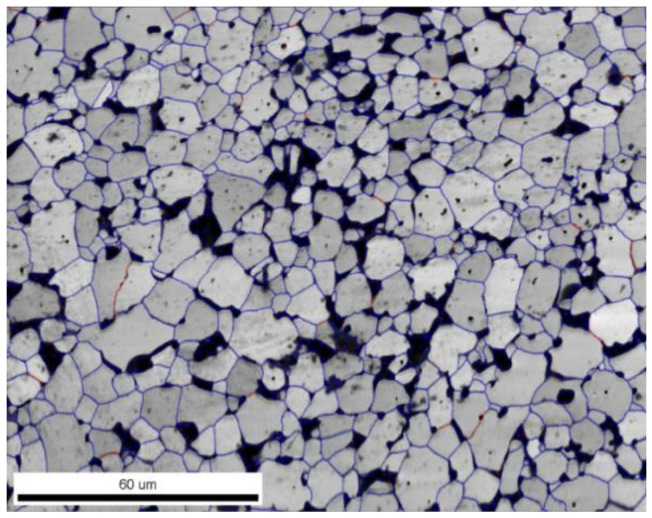
Grain boundaries of the Ti + 20 wt % TiMoC/C composite.

**Figure 6:**
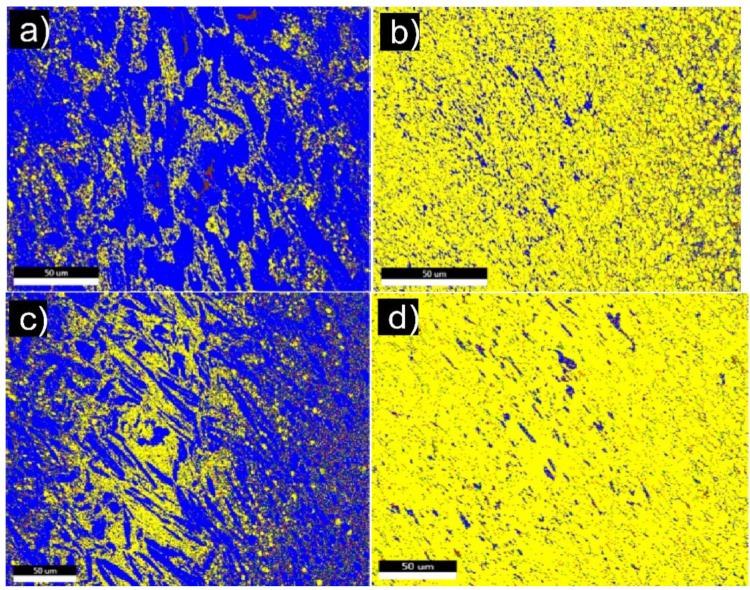
EBSD micrograph comparison of composites: (**a**) Ti + 10 wt %(Ti,Mo)C; (**b**) Ti + 10 wt %(Ti,Mo)C/C; (**c**) Ti + 20 wt %(Ti,Mo)C; (**d**) Ti + 20 wt %(Ti,Mo)C/C.

The amount of HAGB is similar to the Ti + 20 wt % TiMoC composite and is 95.7%. The LAGB in the amount of 4.3% are mostly located between grains. Pores microstructure is quite non-uniform and they are mostly located between grains (Figure 5). Small pores could be found in the TiC grains that reveals different kinetics of vacancy diffusion. The mechanisms of this effect should be examined in the course of further work. EBSD micrographs of phase composition of TiC-Ti based carbides demonstrate a real opportunity to tailor composite microstructure by alloying with (Ti,Mo)C and (Ti,Mo)C/C nanoparticles (Figure 6). It is possible to control phase composition as well as size of carbide grains.

**Figure 7:**
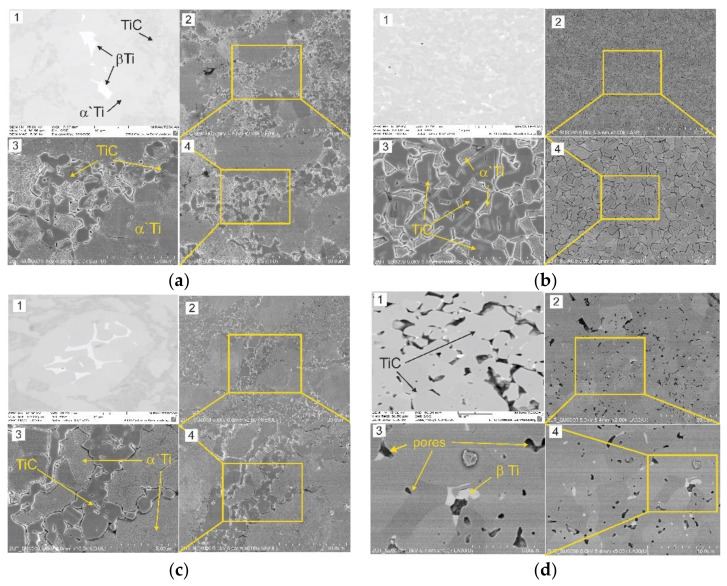
SEM micrographs of the microstructure of the titanium composites: (**a**) Ti + 10 wt %(Ti,Mo)C; (**b**) Ti + 10 wt %(Ti,Mo)C/C; (**c**) Ti + 20 wt %(Ti,Mo)C; (**d**) Ti + 20 wt %(Ti,Mo)C/C. (1) BSE micrograph of a polished specimen, (2–4) SEM micrographs of the composite etched in Fuss reagent by immersion for 30 s.

**Figure 8:**
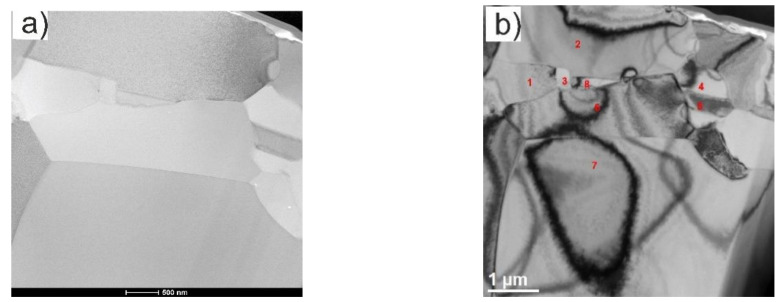
TEM micrographs showing microstructure of the Ti + 20 wt %(Ti,Mo)C composite (the lamella)—surface perpendicular to the direction of composite growth: (**a**) TEM morphology obtained on BF detector; (**b**) TEM micrographs with marked points identified by electron diffraction method; points in the order of numbering from 1 to 8 correspond to the following phases: 1—TiC [031], 2—α-Ti, 3—TiC [211], 4—TiC [100], 5—α-Ti, 6—α’-Ti [010], 7—TiC [211],8—TiC [211].

**Figure 9:**
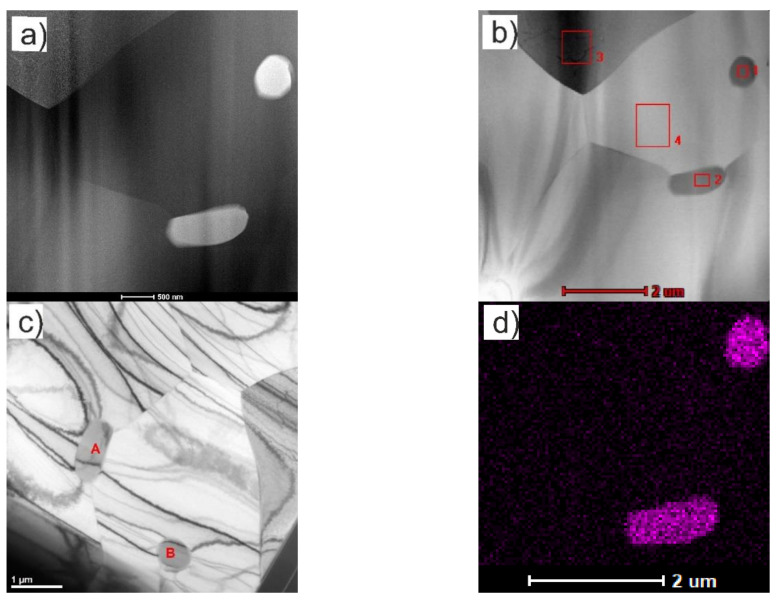
TEM micrographs showing the microstructure of the Ti + 20 wt %(Ti,Mo)C/C composite (the lamella)—surface perpendicular to the direction of composite growth: (**a**–**c**) TEM morphology obtained on HAADF and BF detectors, respectively, with marked points identified by electron diffraction method; points in the order of numbering from 1 to 4 correspond to the following phases: 1,2 correspond to phase β-Ti, (body-centered cubic phases with the lattice parameter a = 0.331 nm), while points 3,4 correspond to phase TiC; (**d**) EDS mapping of the Mo (violet) distribution in the lamella material.

SEM and TEM micrographs of the microstructure of the Ti + (Ti,Mo)C and Ti + (Ti,Mo)C/C composites illustrated in Figure 7, Figure 8 and Figure 9 give evidence for the distribution of reinforcements in the matrix. These observations showed that the strengthening phase consisted of submicro- and microstructured grains. In all tested composites, TiC phase and a two-phase matrix structure containing alpha and beta titanium were identified. In case of Ti + 20 wt %TiMoC/C composite, SEM examinations revealed the presence of pores, which was also confirmed by the lowest relative density of 98%, compared to the above 99%, in the case of other composites.

**Figure 10:**
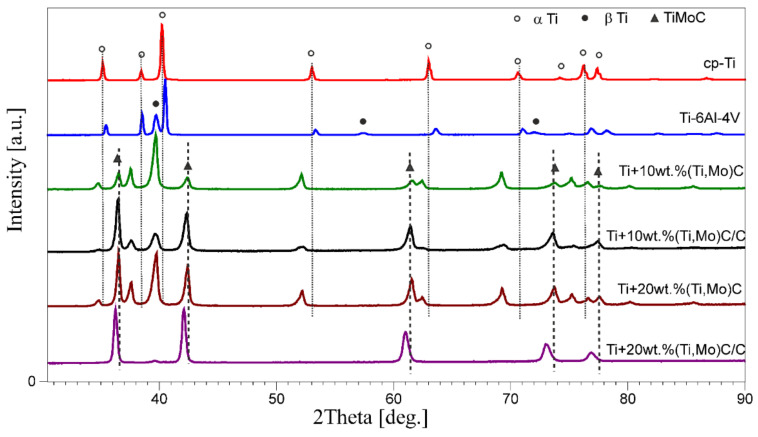
Comparison of the X-ray diffraction patterns of the cp-Ti, Ti6Al4V alloy and Ti + (Ti,Mo)C and Ti + (Ti,Mo)C/C composites after the SPS process.

**Figure 11:**
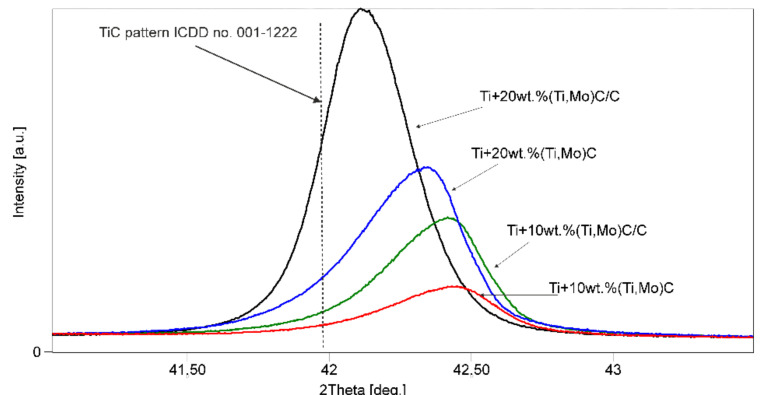
Comparison of the position of interference in reflections (200) of carbides in the TiMMCs.

The X-ray diffraction patterns are shown in Figure 10 and Figure 11. Figure 10 shows X-ray diffraction patterns of the cp-Ti, Ti6Al4V alloy and Ti + (Ti,Mo)C and Ti + (Ti,Mo)C/C composites after the SPS process. In the Ti + 10 wt %(Ti,Mo)C, Ti + 10 wt %(Ti,Mo)C/C and Ti + 20 wt %(Ti,Mo)C composites, α’-Ti and TiC phases were identified by XRD method, while in the Ti + 20 wt %(Ti,Mo)C/C composites, β-Ti(Mo) and TiC and traces of α’-Ti phase were identified.

In the case of the (Ti,Mo)C powder, the analysis of the d_200_ line allows the identification of two sub-networks, probably TiC and MoC (Figure 11). The position of the maximum interference in reflections is not consistent with the maximum line d_200_ of the TiC pattern (ICDD database). In Ti + (Ti,Mo)C composites, the position of the maximum d_200_ line of carbides is shifted towards higher interplanar distances (closer to the TiC pattern). This shift was caused by an increase in the TiC/(TiC + MoC) ratio as a result of the reaction of elemental carbon with the titanium matrix.

A change in the intensity distribution of interference in reflections of the titanium matrix and carbides in the composites (Table 2) has been observed. According to the data in ICDD cards, the I_101_/I_002_ of Ti peak’s ratio is 3.85 (card number 00-005-0682). The I_101_/I_002_ of Ti peak’s ratio is significantly smaller for the Ti + 10 wt %(Ti,Mo)C, Ti + 10 wt %(Ti,Mo)C/C and Ti + 20 wt %(Ti,Mo)C composites analyzed compared to the cp-Ti specimen, for which the value of this ratio is 6.2149. In the case of the Ti + 10 wt %(Ti,Mo)C and Ti + 10 wt %(Ti,Mo)C/C composites, the I_101_/I_002_ of titanium peak’s ratio decreases as the fraction of elemental carbon increases.

With the increase of the mass fraction of TiC in the titanium matrix, the I_101_/I_002_ of Ti peak’s ratio in composite specimens increases slightly from 4.2020 to 4.3310 for 10 and 20 wt % of TiC, respectively. With the increase of the mass fraction of (Ti,Mo)C in the titanium matrix, the I_101_/I_002_ of Ti peak’s ratio in composite specimens decreases from 3.2847 to 2.8244 for 10 and 20 wt % of (Ti,Mo)C, respectively. This means that the cp-Ti sintered by SPS exhibits some texture. In the case of Ti + TiC composites, small changes in orientation of Ti crystallites are observed in comparison with random orientation, whereas bigger changes in orientation of Ti crystallites occur in composites with (Ti,Mo)C.

The phase strengthening the composites, i.e. (Ti,Mo)C crystals show the texture. In the Ti + 10 wt %(Ti,Mo)C/C and Ti + 20 wt %(Ti,Mo)C/C composites, a little lower intensity of the d_111_ diffraction line was observed compared to the d_200_ line, and a little higher in the Ti + 10 wt %(Ti,Mo)C and Ti + 20 wt %(Ti,Mo)C composites. In all the composites, the texture of carbides in the titanium matrix was found. With the increase of the mass fraction of (Ti,Mo)C in the titanium matrix, the I_200_/I_111_ of TiC peak’s ratio in composite specimens increases from 0.8221 to 0.9768 for 10 and 20 wt % of (Ti,Mo)C, respectively, and from 1.0020 to 1.0934 for 10 and 20 wt % of (Ti,Mo)C/C, respectively. The increase in the amount of both carbides and elemental carbon resulted in the reorientation of crystallites towards random orientation.

During the sintering/melting step in the SPS process, the metallic phase forms a liquid in which dissolution and transport of the molybdenum mass takes place. The observed transformations can be explained by the possibility of spinodal decomposition of the nc-(Ti,Mo)C carbide into TiC and metastable MoC carbides [32,33,34]. Under such conditions, the reaction between titanium matrix and MoC occurred according to the equation:

Ti + MoC = TiC + Mo(1)

The change in Gibbs free energy for the reaction Δ*G* = −136.209 kJ∙mol^−1^ at 1300 °C [35]. The resulting molybdenum creates a Ti-Mo alloy, which contributes to the phase transformations of titanium, i.e. α → α’, α’→ β phases during sintering [17,36,37]. It is known that the Mo has high solubility in Ti and larger atomic radius, causing the lower Young’s modulus of the material matrix, and the presence of larger atomic radius reduces the binding force of the Ti lattice, expanding the unit cell volume [38].

As mentioned above, the reaction between titanium and elemental carbon from shell, took place under SPS process conditions. Comparison of the intensities of interference reflections of carbides and titanium of the matrix shows that the titanium matrix quantitatively dominates in the Ti + 10 wt %(Ti,Mo)C and Ti + 20 wt %(Ti,Mo)C composites, while in the Ti + 10 wt %(Ti,Mo)C/C and Ti + 20 wt %(Ti,Mo)C/C composites, the carbides quantitatively prevail in comparison to the metal matrix phases. The amount of 40 wt % of free carbon in (Ti,Mo) C/C powder, causes loss of titanium in the matrix during the consolidation process. This loss is multiplied by the proportion of the powder introduced in the strengthening phase according to the reaction:

Ti + C = TiC(2)

Additionally, the increase of titanium carbide may be caused by carbothermal reduction of TiO_2_ (passive layer on Ti powder) in the following reaction:

TiO_2_ + 2C = TiC + CO_2_ (3)

The phenomena presented above contribute to significant fragmentation of the nanocomposite microstructure, obtaining good wettability of carbides by the matrix material, elimination of passive TiO_2_ layers from the surface of titanium and carbides, and as a consequence achieving the effect of maximization of interfacial bond strength in TiMMCs.

Roughness plays an important role in determining how a real object will interact with its environment. Some irregularities on the surface may form nucleation sites for cracks or corrosion. In Table 3, the average values of surface roughness (*R_a_*) of the tested materials (specimens) after standard surface treatment described above are presented.

The lowest roughness values were found for Ti-6Al-4V alloy and the Ti + 10 wt %(Ti,Mo)C/C composite, the highest values were observed for Ti + 20 wt %(Ti,Mo)C/C due to surface porosity. Except for the porous specimen of the Ti + 20 wt %(Ti,Mo)C/C composite, the surface of the other materials was characterized by a roughness below 100 nm.

**Figure 12:**
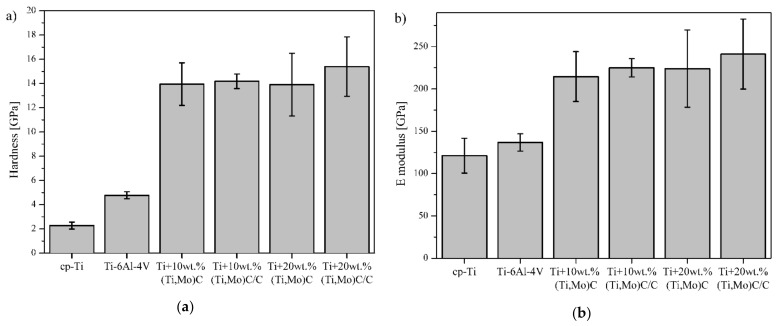
(**a**) Hardness and (**b**) Young’s modulus of the composites with respect to the reinforcing quantity of (Ti,Mo)C/C and reference specimens cp-Ti and Ti-6Al-4V.

**Figure 13:**
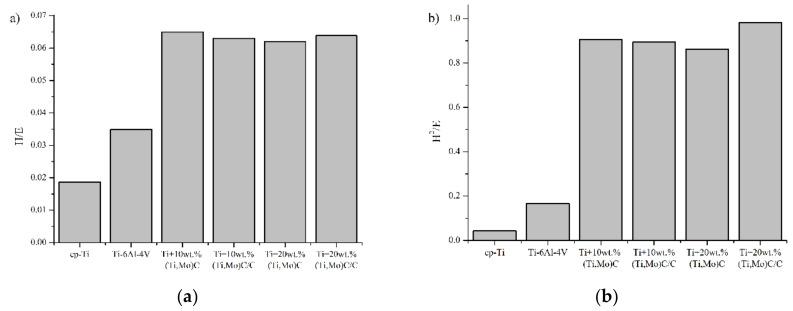

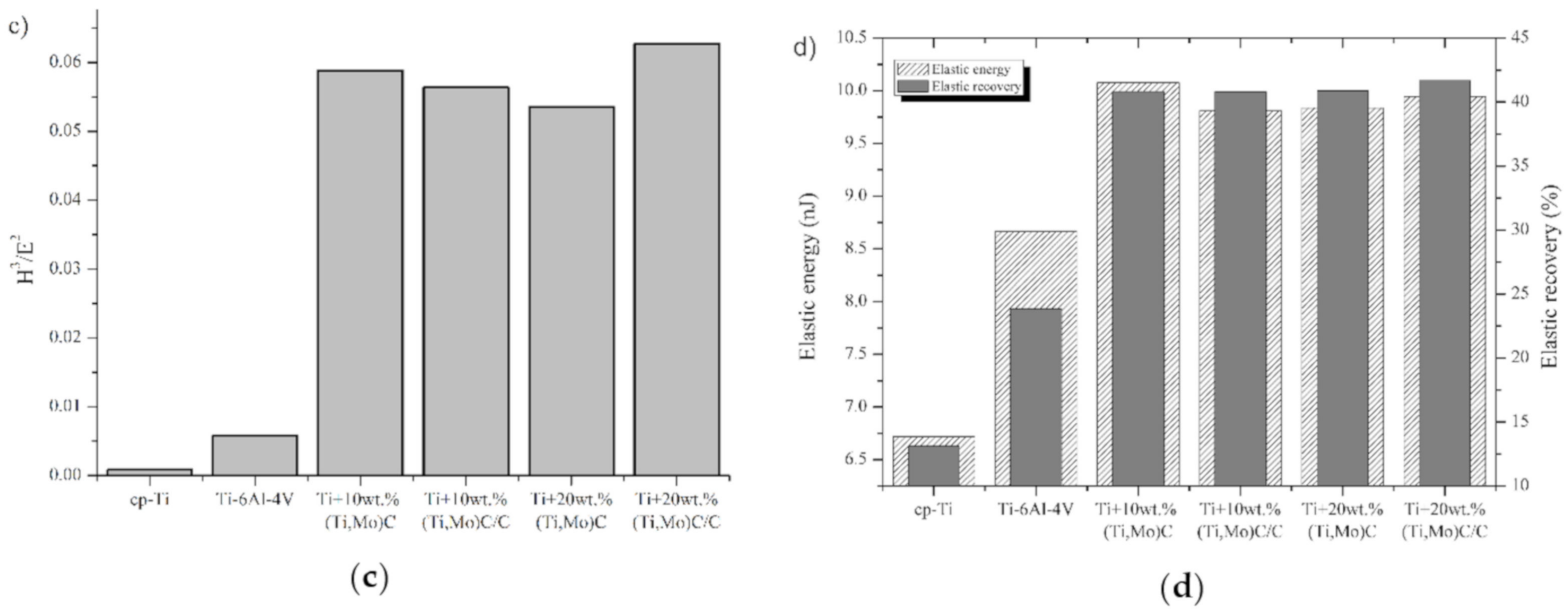
(**a**) H/E; (**b**) H^2^/E, (**c**) H^3^/E^2^; (**d**) elastic energy and elastic recovery values of the composites with respect to reinforcing quantity of the carbides and reference specimens cp-Ti and Ti-6Al-4V.

Figure 12 presents the mechanical properties of the specimens. The highest values were observed for reinforcements (Ti,Mo)C embedded in carbon shell. *E* modulus has a similar trend to hardness. The addition of hard ceramic particles of nc-(Ti,Mo)C into titanium matrix improves mechanical properties. A composite with 10 wt % of (Ti,Mo)C showed significant increase in hardness from 2.3 ± 0.3 GPa (cp-Ti) to 14.2 ± 0.6 GPa. An increase of (Ti,Mo)C phase up to 20% results in a further enhancement of hardness. However, it is well-known that the relationship between hardness (*H*) and Young’s modulus (*E*) is an important variable to describe mechanical properties (Figure 13). In the literature, various authors have suggested *H*/*E* (elastic strain to failure and fracture toughness), *H^2^*/*E* (resistance to permanent deformation) and *H^3^*/*E^2^* (plastic deformation under load) ratios [39].

**Figure 14:**
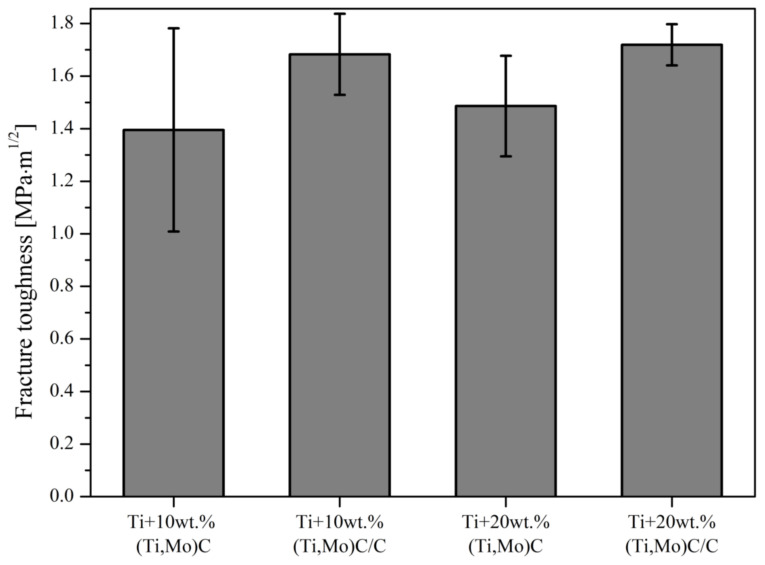
Fracture toughness of the composites with respect to the reinforcing quantity of (Ti,Mo)C and (Ti,Mo)C/C.

Elastic energy and elastic recovery of the composites are at a comparable level but take higher values compared to the Ti-6Al-4V alloy and cp-Ti. Hardness as a parameter for wear resistance is not sufficient for evaluation of wear and crack resistance. Musil et al. [40] suggested that materials with a high ratio *H/E* > 0.1 and low *E* modulus are required for the development of hard materials with enhanced toughness. The addition of hard nc-(Ti,Mo)C particles to the titanium matrix significantly increases the *H*/*E* value. Composite with 20 wt % of (Ti,Mo)C/C revealed a significant increase of *H*/*E* from 0.019 to 0.064. Fracture toughness was calculated with the radial indentation crack method using Vickers hardness tester (HV_1_) with a dwell time of 15 s (Figure 14).

Addition of the nc-(Ti,Mo)C/C particles to the titanium matrix will increase the resistance fracture toughness of the titanium composites. Specimens Ti + 10%(Ti,Mo)C/C and Ti + 20%(Ti,Mo)C/C have the highest values of fracture toughness, whereas composites reinforced with particles of the (Ti,Mo)C type show lower values. Fracture toughness of the composite is subject to a measurement error due to its porosity. These results reveal that the (Ti,Mo)C reinforcements embedded in carbon shell slightly increase fracture toughness of the titanium composites. At the same time, there is practically no difference between 10 and 20%.

**Figure 15:**
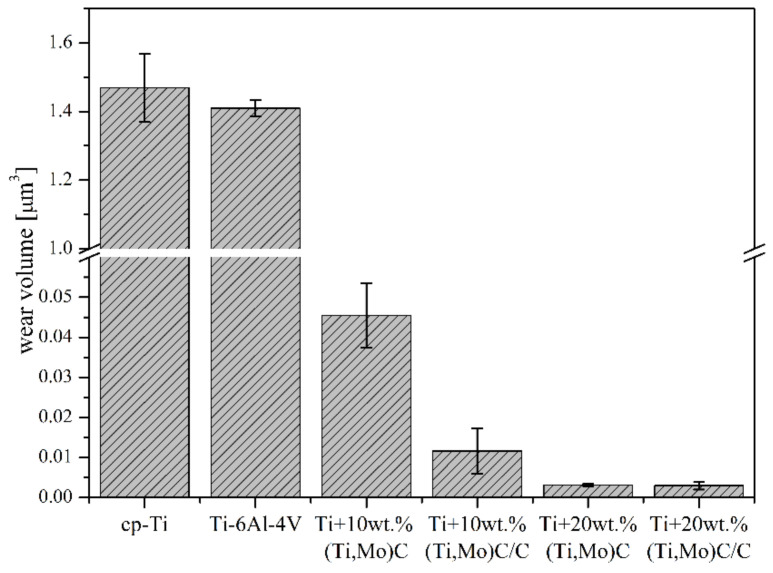
Wear volume of the composites with respect to reinforcing quantity of reference (Ti,Mo)C/C.

The materials obtained under the SPS process were subjected to tribological tests and wear volumes were determined (Figure 15). The lowest values of wear volume of the titanium composites were observed for Ti + 20 wt %(Ti,Mo)C and Ti + 20 wt %(Ti,Mo)C/C, the highest for Ti + 10 wt %(Ti,Mo)C. Reference specimens of Ti6Al4V alloy and cp-Ti showed significant wear in comparison with composites, their wear volume reached ca. 1400–1500 times higher value. Addition of hard ceramic particles of nc-(Ti,Mo)C and nc-(Ti,Mo)C/C into Ti matrix improve wear properties. When the contribution of the ceramic phase increases, the wear coefficient decreases.

**Figure 16:**
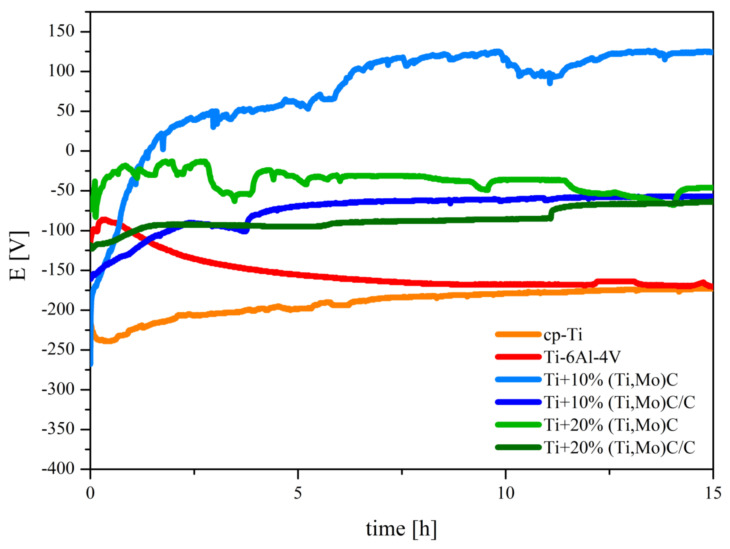
The curves of OCP variation versus time of the specimens exposition to the 3.5 wt % NaCl solution at temperature 22–23 °C.

**Figure 17:**
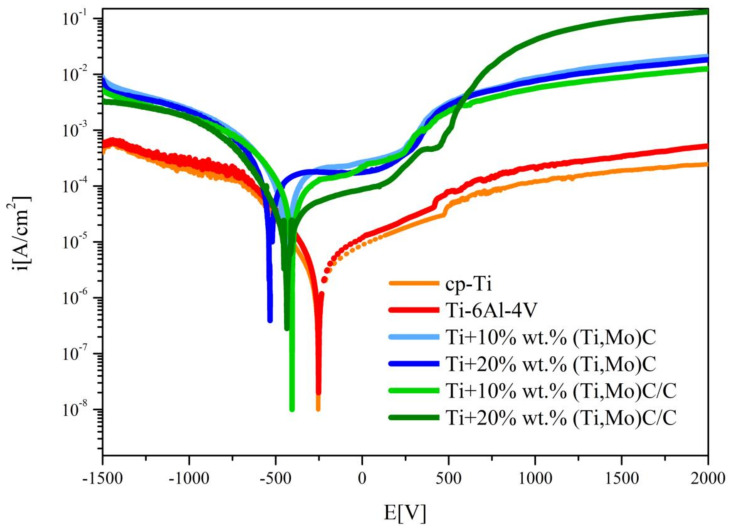
Comparison of the anodic polarization curves of the composites, cp-Ti, and Ti-6Al-4V alloy.

The comparison of the results of corrosion tests of the Ti + (Ti,Mo)C and Ti + (Ti,Mo)C/C composites and reference specimens of cp-Ti and Ti-6Al-4V alloy are shown in Figure 16 and Figure 17. The variation in the OCP of the composites exposed to a 3.5 wt % NaCl solution at RT after polishing is presented in Figure 16. It is observed from the OCP profiles of the Ti + 10 wt %(Ti,Mo)C composites that they achieved the highest value in comparison with other compositions. This potential reached positive values after 1 h, then fluctuated until it stabilized after about 12 h. The OCP of the Ti + 20 wt %(Ti,Mo)C composite initially reached its highest values, then it fluctuated and stabilized at a level comparable to that of the Ti + 10 wt % (Ti,Mo)C/C, and Ti + 20 wt % (Ti,Mo)C/C composites. The observed fluctuations were probably caused by surface defects of the above-mentioned composites and the related tendency to local corrosion. The lowest OCP values were reached for cp-Ti and Ti-6Al-4V. By comparing titanium composites, it can be concluded that composites characterized by lower content of elementary carbon in sintered powders tend to achieve the highest values of OCP potentials.

In case of the cp-Ti specimen immersed in 3.5 wt % NaCl solution, corrosion potential was at the level of −0,251 V and corrosion current density reached 0.7 × 10^−6^ A·cm^–2^, and in the case of Ti-6Al-4V alloy, corrosion potential was at the level of −0.252V and corrosion current density reached 1.2∙10^−6^ A·cm^–2^ (Table 4).

The values of the corrosion potentials of the composites in comparison to the reference materials, i.e. cp-Ti and Ti-6Al-4V, are about 200–250 mV lower and the densities of the corrosion currents are about 10-30 times higher (Figure 17). When TiMMCs are immersed in 3.5 wt % NaCl solution, at RT 22–23 °C, the corrosion potential generally shifts to lower values towards the cathodic region. Composites of Ti + Ti,Mo)C/C type showed more favorable corrosion properties in comparison to Ti + (Ti,Mo)C. The values of the corrosion current density of Ti + (Ti,Mo)C/C composites are about 2–3 times lower compared to Ti + (Ti,Mo)C. Moreover, higher values of corrosion potential were found in the case of composites with lower carbide content in the matrix. The best corrosion resistance, measured by the relatively higher values of resistance polarization (R_pol_), characterized the Ti-6Al-4V alloy, pure titanium, and the Ti + 20 wt %(Ti,Mo)C/C composite. By comparing titanium composites, it can be concluded that the addition of carbon to the Ti + (Ti,Mo)C composites increases their resistance to electrochemical corrosion.

The corrosion mechanism in titanium, titanium alloy, and Ti-based MMCs is mainly attributed to the fact that compact and stable oxide passive films can be formed on the material surface. 

**Figure 18:**
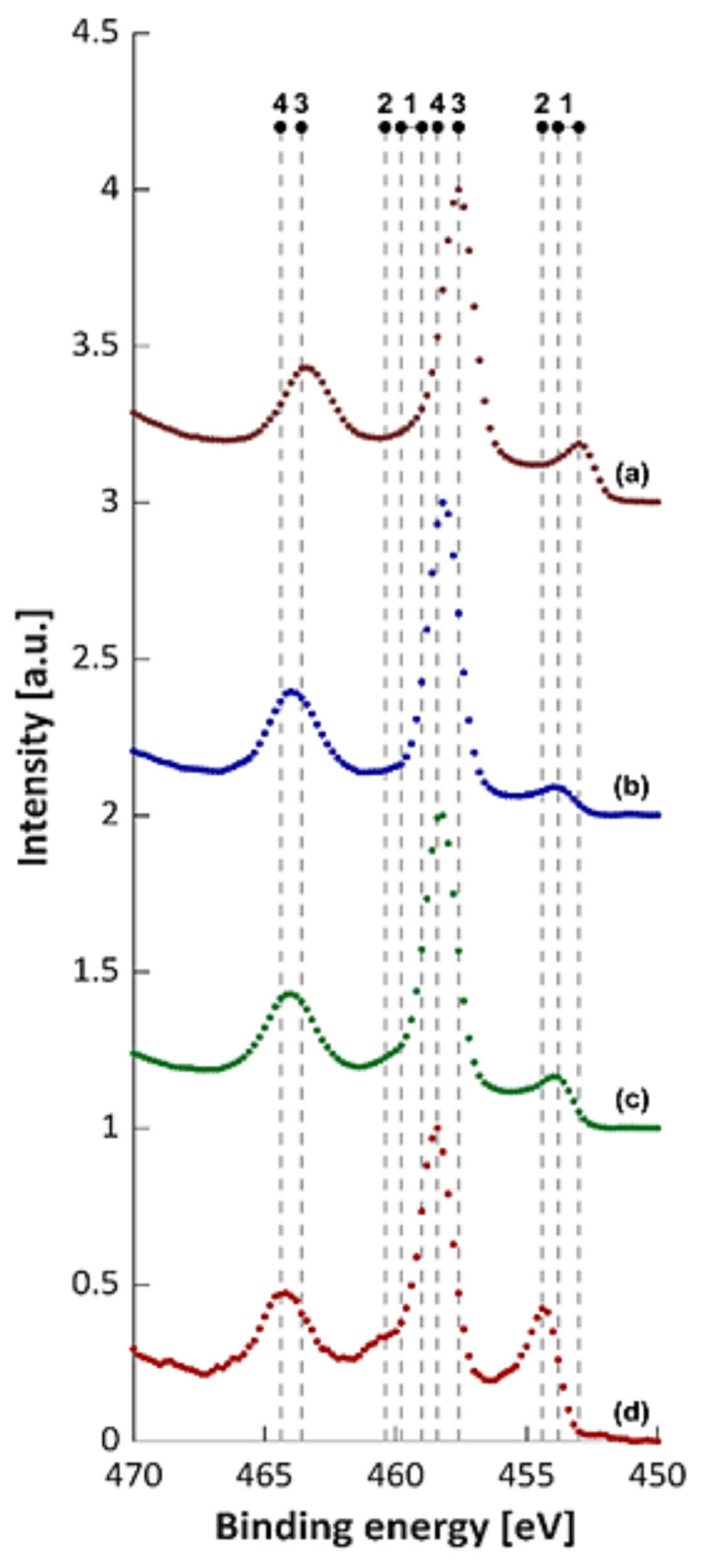
XPS spectra of Ti2p3/2: 1—453.0–453.8 eV, (Ti0), 2—454.4 eV (TiC), 3—458.4 eV (TiO2), 4—458.6 eV (TiO2 doped Mo6+). (**a**) Ti + 10 wt %(Ti,Mo)C, (**b**) Ti + 10 wt %(Ti,Mo)C/C, (**c**) Ti + 20 wt %(Ti,Mo)C, (**d**) Ti + 20 wt %(Ti,Mo)C/C.

The Ti + (Ti,Mo)C composites have also been inspected by XPS analysis. Figure 18 displays example of Ti 2p_3/2_ and Ti 2p_1/2_ spectra before etching recorded at binding energies in the range 450–470 eV. The analysis of Ti2p_3/2_ indicates presence of TiC (454.4 eV) as well as TiO_2_ (458.4 eV) and doped with molybdenum TiO_2_-Mo^6+^ (458.4 eV) and small contribution of Ti^0^ [41].

The Ti 2p spectrum of the Ti + 10 wt %(Ti.Mo)C/C composite reveals the highest percentage of TiO_2_ phase, while in the case of the Ti + 20 wt %(Ti.Mo)C/C composite, the highest percentage of TiC phase. The Ti 2p spectrum of the Ti + 10 wt %(Ti.Mo)C/C and Ti + 20 wt %(Ti.Mo)C/C composites reveals remains of TiO_2_ layer, whose lines are shifted by about 0.2 eV towards higher values of bond energy in comparison to the pure TiO_2_. This may be related to Mo admixtures in the TiO_2_ structure [42].

**Figure 19:**
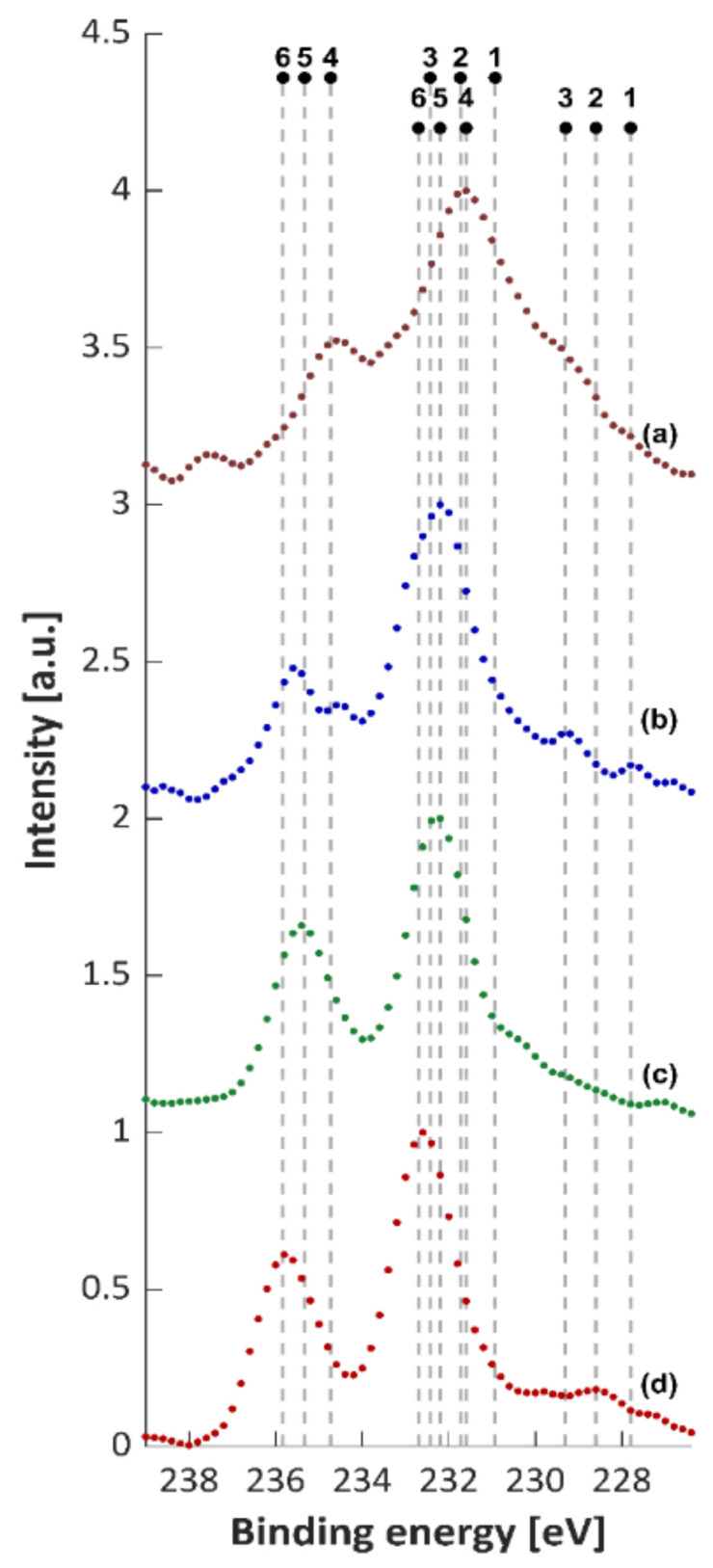
XPS spectra of Mo3d5/2: 1—227.8 eV (Mo0), 2—228.6 (Mo2+), 3—229.3 (Mo4+, MoO2), 4—231.6 eV (Mo5+; MoO3), 5—232.2 eV (Mo6+), 6—232.7 eV (Mo6+; MoO3). (**a**) Ti + 10 wt %(Ti,Mo)C, (**b**) Ti + 10 wt %(Ti,Mo)C/C, (**c**) Ti + 20 wt %(Ti,Mo)C, (**d**) Ti + 20 wt %(Ti,Mo)C/C.

Figure 19 displays the XPS spectra of the Mo3d before etching. According to the deconvolution results, the Ti 2p spectrum of the composites is dominated by species in the Mo^6+^ oxidation state, mainly MoO_3_ with the presence of a small contribution of 5^+^, 4^+^ and 2^+^. The subcomponents detected by peak fitting at 229.3 eV and at 231.6 eV are attributed to Mo 3d_5/2_ of MoO_2_, and of Mo_2_O_5_, respectively [43,44,45]. The existence of a noticeable percentage of MoO_2_ and Mo_2_O_5_ oxides on the surface of the composite together with the Mo^0^ phase distinguishes the Ti + 10 wt % (Ti, Mo)C and Ti + 10 wt % (Ti, Mo)C/C composites.

In all the composites in the passive layer, MoO_3_ was identified. The composition of passive layer are given in Table 5. It is likely that MoO_3_ and TiO_2_ oxides show mutual solubility and are responsible for corrosion resistance of the composites. Composite Ti + 10 wt %(Ti,Mo)C, which reached the highest value of OCP potential, differs from the other composites by a higher oxide layer thickness and higher percentage of Mo_2_O_5_, and MoO_2_ type oxides. It can be concluded that the dominant phase in the passive layer of composites is TiO_2_ doped with MoO_3_. The microstructure of this layer influences the corrosion resistance of titanium composites as well as Ti-Mo alloys [46].

## 4. Conclusions

The subject of the research was titanium composites obtained by the SPS technique in the reactive sintering of a mixture of microcrystalline titanium powders and nanocrystalline (Ti,Mo)C powders in carbon shells at 1300 °C. The percentage of carbon in the carbon shells was ca. 3 or 40 wt % of powders of the (Ti,Mo)C/C type. Sintered compacts of cp-Ti and Ti6Al4V alloy were produced as reference specimens under the same conditions. The aim of these studies was an attempt to explain the influence of carbon on the morphology, microstructure, and mechanical and corrosion properties of these composites. The research showed that composites with a two-phase matrix microstructure (α’-Ti and β-Ti (Mo)) reinforced with submicro- and microstructural TiC carbide have been obtained. EBSD examination results of the TiC-Ti based carbides demonstrate a real opportunity to tailor composite microstructure by alloying with (Ti,Mo)C and (Ti,Mo)C/C nanoparticles. It is possible to control both phase composition and size of carbide grains. The composites manufactured from the Ti + (Ti,Mo)C/C type mixtures were characterized by the highest wear resistance in the friction process compared to the Ti + (Ti,Mo)C composites and cp-Ti and the Ti6Al4V alloy. A higher percentage of carbon in the carbon shell—approx. 40 wt %—also contributed to increased fracture toughness. All titanium composites were characterized by higher values of the OCP potential compared to cp-Ti and Ti6Al4V alloy. The passive layers, mainly consisting of TiO_2_ doped with MoO_3_ oxide with the presence of a small contribution of MoO_2_ and Mo_2_O_5_ oxides, are responsible for these properties. Compared to titanium and its alloy, titanium composites showed a higher corrosion rate and higher passivation currents under polarization conditions in a 3.5% NaCl solution.

## Figures and Tables

**Table 1:** Percentage of the identified phases.

#	Composite Type	Phase List			
		TiC, wt %	α-Ti, wt %	β-Ti, wt %	Mo, wt %
1	Ti + 10 wt % (Ti,Mo)C	17	76	3	4
2	Ti + 10 wt %(Ti,Mo)C/C	79	14	3	4
3	Ti + 20 wt % (Ti,Mo)C	26	59	6	9
4	Ti + 20 wt %(Ti,Mo)C/C	83	1	7	9

**Table 2:** Changes in intensity of diffraction peaks in XRD spectra of the TiMMCs.

Material	I_101_/I_002_ α-Ti	Material	I_200_/I_111_ Carbides
cp-Ti	6.2149	TiMoC powder	1.7611
Ti + 10 wt %(Ti,Mo)C	3.2847	TiMoC/C powder	1.5402
Ti + 10 wt %(Ti,Mo)C/C	2.4456	Ti + 10 wt %(Ti,Mo)C	0.8221
Ti + 20 wt %(Ti,Mo)C	2.8244	Ti + 10 wt %(Ti,Mo)C/C	1.0020
Ti + 20 wt %(Ti,Mo)C/C	At the limit of detection in the XRD method	Ti + 20 wt %(Ti,Mo)C	0.9768
		Ti + 20 wt %(Ti,Mo)C/C	1.0940

**Table 3:** The average roughness (*R_a_*) of the cp-Ti, Ti-6Al-4V alloy and the titanium composites.

Material	Roughness (nm)	Standard Devation (nm)
cp-Ti	71.55	38.90
Ti-6Al-4V	23.18	17.52
Ti+10wt%(Ti,Mo)C	45.03	32.33
Ti+10wt%(Ti,Mo)C/C	22.67	13.78
Ti + 20 wt %(Ti,Mo)C	51.45	21.29
Ti + 20 wt %(Ti,Mo)C/C	213.27	203.95

**Table 4:** Corrosion parameters of the composites exposed to 3.5 wt % NaCl solution RT.

Material	E_kor,_ (V)	i_cor_ (A/cm^2^)	R_pol_ (Ω/cm^2^)	B_a_ (V)	B_c_ (V)
cp-Ti	−0.254	0.40 × 10^−6^	29.1 × 10^3^	0.047	0.054
Ti6Al4V	−0.252	0.40 × 10^−6^	21.6 × 10^3^	0.039	0.042
Ti + 10%(Ti,Mo)C	−0.425	10.30 × 10^−6^	0.9 × 10^3^	0.041	0.044
Ti + 10%(Ti,Mo)C/C	−0.405	6.20 × 10^−6^	1.2 × 10^3^	0.035	0.033
Ti + 20%(Ti,Mo)C	−0.533	5.78 × 10^−6^	0.9 × 10^3^	0.021	0.030
Ti + 20%(Ti,Mo)C/C	−0.433	2.40 × 10^−6^	3.3 × 10^3^	0.038	0.035

**Table 5:** Chemical composition of the passive layer.

#	Material	Ti (at.%)	Mo (at.%)	C (at.%)	O (at.%)	Mo/Ti	C/Ti	O/Ti
1	Ti + 10%TiMoC	9.2	0.3	61.3	29.2	0.01	6.66	3.17
2	Ti + 10%TiMoC/C	11.5	0.2	49.9	38.4	0.02	4.34	3.34
3	Ti + 20%TiMoC	14.4	0.6	46.4	38.6	0.04	3.22	2.68
4	Ti + 20%TiMoC/C	13.2	0.2	49.9	36.6	0.02	3.78	2.77

## Data Availability

Data sharing is not applicable to this article.
